# Egg Consumption and Stroke Risk: A Systematic Review and Dose-Response Meta-Analysis of Prospective Studies

**DOI:** 10.3389/fnut.2020.00153

**Published:** 2020-09-08

**Authors:** Hui Tang, Yi Cao, Xiang Yang, Yuekang Zhang

**Affiliations:** ^1^Department of Neurosurgery, West China Hospital, Sichuan University, Chengdu, China; ^2^Department of Neurosurgery, Nanchong Central Hospital, The Second Clinical Medical College, North Sichuan Medical College, Nanchong, China

**Keywords:** egg consumption, stroke risk, systematic review, meta-analysis, dose-response

## Abstract

**Background:** The present study was performed to systematically quantify the association between egg consumption and stroke risk as inconsistent results have been produced.

**Methods:** Three electronic databases (PubMed, Embase, and Cochrane Library), previous reviews, meta-analyses, and bibliographies of relevant articles were retrieved from prospective cohort studies published before July 1, 2020. The random-effects model was employed to estimate summary relative risks (RRs) and 95% confidence intervals (CIs). A dose-response analysis was also performed when data were available.

**Results:** Sixteen publications involving 24 prospective cohort studies were included in our final meta-analysis. No significant association between egg consumption and stroke risk was identified (RR = 0.92, 95% CI: 0.84–1.01) for the highest vs. the lowest quintiles of egg intake. Subgroup analysis indicated that geographic location significantly modified the effect of egg consumption on stroke risk. Higher egg consumption was attributed to a reduced probability of stroke in Asia (RR = 0.83, 95% CI: 0.73–0.94), but not in North America (RR = 0.95, 95% CI: 0.77–1.16) or Europe (RR = 1.02, 95% CI: 0.91–1.16). Dose-response analysis demonstrated a nearly J-shaped curve between egg consumption and risk of stroke. A decreased risk was observed for the intake of one to four eggs weekly and an increased risk for the intake of more than six eggs weekly. The results were significant at an intake of 10 eggs weekly.

**Conclusions:** The evidence from this meta-analysis showed that a J-shaped association exists between egg consumption and stroke risk.

## Introduction

Stroke is the major cause of death and disability in most regions globally (1). Modifiable factors, including metabolic factors (high systolic blood pressure, body mass index, fasting plasma glucose, and cholesterol), behavioral factors (smoking, poor diet, and low physical activity), and environmental factors (air pollution and lead exposure), play essential roles in the development of stroke, with the exception of non-modifiable factors (age, sex, race, heredity, and personal history of prior stroke or cardiovascular disease) (1). Over the past few decades, egg consumption has garnered considerable attention in relation to public health, as the multifaceted effect of egg intake has been described (2, 3). The fairly low-calorie egg is a rich source of high-quality protein, folate, choline, riboflavin, selenium, and many vitamins but also has a high dietary cholesterol level. Epidemiological studies focusing on the association between egg consumption and stroke have produced inconsistent results (4–22). Five studies showed an increased risk of stroke (5, 10, 12, 16, 18, 22), although the results were not statistically significant, some indicated a significant or non-significant inverse relationship (6–8, 17, 20, 21), and only one study revealed no association (14). To provide a reliable quantitative assessment of this association, several meta-analyses were performed (19, 20, 23–26). Previous studies mainly involved the relationship between total stroke and egg consumption, but few investigations clarified the effect of sex, stroke type, dose-response on stroke risk, and regional difference. Thus, questions regarding the strength and shape of the dose-response relationship have yet to be answered. Therefore, we performed an updated meta-analysis of prospective cohort studies following the proposal for reporting systematic reviews and meta-analysis (PRISMA statement) (27).

## Methods

### Search Strategy

The PubMed, Embase, and Cochrane Library databases were searched up to July 1, 2020. Details of the search terms are listed in Supplementary Table 1. To identify additional relevant studies, the bibliographies of qualifying articles were searched manually. If necessary, an email was sent to the corresponding author of interest.

### Eligibility Criteria

Only prospective cohort studies on the association between egg consumption and stroke risk were included. The endpoint was fatal and/or non-fatal stroke cases. Adjusted relative risk (RR) estimates including hazard ratios or risk ratios with 95% confidence intervals (CIs) should be reported in the original studies. When multiple reports from the same cohort study were identified, the studies with the longest follow-up time were included.

### Data Extraction

The first author's name, publication year, name of cohort study, sex, and age of the population at baseline, years of follow-up, number of cases and cohort size, endpoint of cases, method of exposure assessment, the fully adjusted risk estimates with 95% CIs for the corresponding level of egg intake, and confounding factors were extracted from the selected studies. Any discrepancies were resolved by discussion.

### Study Risk-of-Bias Assessment

The risk of bias in the included studies was evaluated by the nine-star Newcastle–Ottawa Scale (NOS) (28). In this meta-analysis, we adopted this guideline with some modifications. In particular, regarding exposure ascertainment, one star was assigned to a cohort study in which the exposure data were provided at baseline and updated during follow-up. According to the NOS guideline, a maximum of two stars can be assigned for comparability. As confounding factors are the major concern in observational studies, no more than one star could be assigned. When the included studies provided adjusted risk estimates, one star was assigned. Otherwise, no star was assigned. For the duration of follow-up, one star was assigned to a cohort study with 10 follow-up years or more. We assumed that a study with ≥7 stars was considered to be of high quality, as there are no established standards.

### Grading Quality of Evidence

The NutriGrade scoring system was adopted to judge the meta-evidence (29). The NutriGrade scoring system comprises eight subitems as follows: risk of bias, study quality, and study limitations (maximum of 2 points), precision (maximum of 1 point), heterogeneity (maximum of 1 point), directness (maximum of 1 point), publication bias (maximum of 1 point), funding bias (maximum of 1 point), effect size (maximum of 2 points), and dose-response (maximum of 1 point). Based on this scoring system, the level of meta-evidence was categorized as high (≥8), moderate (6–7.99), low (4–5.99), and very low (0–3.99).

### Statistical Methods

We calculated the summary RRs of stroke for the highest vs. the lowest levels of egg intake. When separate risk estimates for males, females, and stroke subtypes were available in a study, we divided it into separate studies. One study did not use the lowest as the reference category (5), and we calculated the corresponding estimates of the highest vs. the lowest levels of egg intake using the method proposed by Hamling et al. (30). A random-effects model was adopted (31), as heterogeneity across studies was considered. Heterogeneity between studies was assessed with *Q* (significance level at *p* < 0.10) and *I*^2^ statistics (32). Values of 25, 50, and 75% represent mild, moderate, and severe heterogeneity, respectively. Subgroup analyses were performed according to the geographic region, sex, stroke subtypes, and study quality. Sensitivity analyses were conducted by excluding one study in turn to assess the robustness of the overall result. Potential publication bias was evaluated by visual inspection of Begg's funnel plots and the Egger linear regression test (significance level at *p* < 0.05) (33, 34).

As the levels of egg consumption were heterogeneous, 50 g was defined as a serving size or one egg, unless a serving size was specified in the original paper. To understand the shape of the dose-response relationship, a dose-response analysis was conducted with a one-stage robust error meta-regression model (REMR), based on inverse variance weighted least squares regression and cluster-robust error variances (35). With this method, the distribution of cases or person-years was not required, but risk estimates with 95% CIs for at least two levels of egg exposure should be reported. The midpoint was calculated for studies that reported a range of intake and did not provide the mean or median intake for the range of egg intake. When the highest level of egg intake was open-ended, it was assumed that the exposure category was set at 1.2 times the lower boundary. In our study, the dose-response association between stroke risk and egg consumption (g/week) was reported.

All analyses were performed using STATA statistical software (version 15.0, STATA Corp., College Station, TX, USA).

## Results

### Literature Search

**Figure 1:**
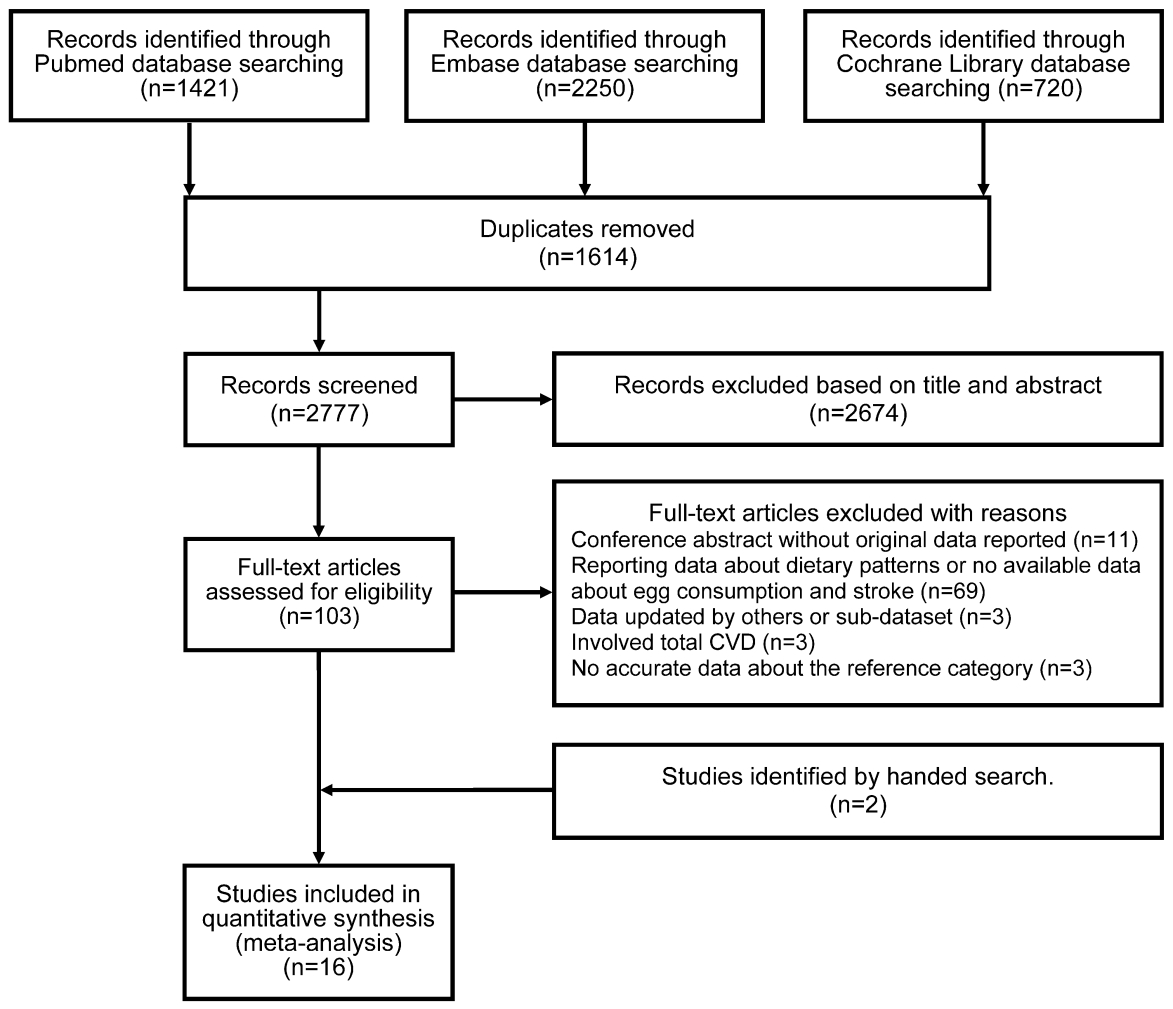
PRISMA flow diagram of the literature search and study selection.

The PRISMA flow diagram illustrates the selection of included studies and the screening process in this study (Figure 1). The initial search identified 4,391 articles from PubMed, Embase, and Cochrane Library. Of these articles, 1,614 were duplicates, and 2,674 articles were excluded at the first screening stage after review of the titles and abstracts as they were considered irrelevant. Following full-text assessment, 89 of 103 potentially relevant studies were further removed after application of the inclusion and exclusion criteria. The reasons for excluding articles were categorized into five groups and are listed in Supplementary Table 2. Two cohort studies were identified by a manual search (5, 15). Ultimately, 16 articles involving 24 cohort studies were included (4–9, 12, 13, 15–22).

### Basic Characteristics

Table 1 summarizes the basic characteristics of the studies. All individuals were aged 17 years or above at baseline. Nine studies presented gender-specific results (5, 7–9, 13, 16, 17, 19, 21). Seven studies were from the USA (6–9, 12, 18, 19), two from China (17, 20), two from Japan (4, 5), and one each from Iran (15), Finland (21), the United Kingdom (16), and Sweden (13). A majority of the included studies reported fatal stroke cases as the endpoint of interest (4–6, 8, 15–17, 19, 20), while others reported both fatal and non-fatal cases. Ascertainment of stroke outcome was not entirely consistent across the studies and included death certificate registers, medical records, hospital discharge, and self-report with validation. An interviewer-administered or self-administered food frequency questionnaire (FFQ) was employed to assess egg consumption, but in most of the included studies, the FFQ was collected at baseline (4–8, 12, 13, 15, 18–22). With regard to confounding factors adjusted in the original studies, all the included studies adjusted age and sex in the basic model. In the multivariate model, various confounding factors were considered, mainly personal characteristics (body mass index, education level, and marital status), history of personal disease (diabetes, hypertension, hypercholesterolemia, and cardiovascular disease), drug use, and dietary lifestyles (smoking, alcohol consumption, physical activity, and the intake of fruit, vegetables, meat, and others). A total of 12 studies were awarded more than six stars (4, 5, 9, 12, 13, 15–19, 21, 22), and four studies obtained six stars (5, 7, 8, 20).

**Table 1:** Main characteristic of included studies.

**References**	**Location**	**Study name**	**Sex**	**Age at baseline**	**Follow-up year**	**No. of cases**	**No. of participants**	**Endpoint**	**Exposure Assessment**	**Risk of bias (✩)**
Sauvaget et al. (4)	Japan	LSS	Both	34–103	16	1,462	37,130	Stroke mortality	Interviewer-administered FFQ at baseline	7
Nakamura et al. (5)	Japan	NIPPON DATA80	Both	≥30	14	219	9,263	Stroke mortality	Self-administered FFQ at baseline	6
Qureshi et al. (6)	USA	NHANES-I	Both	25–74	20	655	9,734	Stroke mortality	Interviewer-administered FFQ at baseline	7
Djoussé et al. (7)	USA	PHS	Male	40–85	20	1,342	21,327	Total Stroke	Self-administered FFQ at baseline	6
Scrafford et al. (8)	USA	NHANES III	Both	17+	8.8	137	14,946	Stroke mortality	Self-administered FFQ at baseline	6
Goldberg et al. (12)	USA	NOMAS	Both	>40	11	266	2,429	Total stroke,	Interviewer-administered FFQ at baseline	7
Larsson et al. (13)	Sweden	CSM, SMC	Both	<60	13	3651	70,571	Total stroke,	Self-administered FFQ at baseline	7
Farvid et al. (15)	Iran	GCS	Both	36–85	11	507	42,403	Stroke mortality	Interviewer-administered FFQ at baseline	7
Guo et al. (16)	The UK	CAPS	Male	45–59	22.8	248	2,512	Stroke mortality	Interviewer-administered FFQ at baseline, updated every 5 years	8
Qin et al. (17)	China	CKB	Both	30–79	8.9	34,823	461, 213	Total stroke, stroke mortality	Interviewer-administered FFQ, at baseline, second and third survey	7
Xu et al. (20)	China	GBCS	Both	≥50	9.8	341	28,024	Stroke mortality	Interviewer-administered FFQ at baseline	6
Mazidi et al. (19)	USA	NHANES 1999–2000	Both	≥20	11	NR	23,524	Stroke mortality	Interviewer-administered FFQ at baseline	7
Zhong et al. (18)	USA	ARIC, CARDIA, FHS, FOS, JHS, MESA	Both	51.6 ± 13.5	17.5	1,302	29,615	Total Stroke	interviewer-administered FFQ at baseline	7
Abdollahi et al. (21)	Finland	KIHD	Male	42–60	21.2	217	1,015	Total stroke	Interviewer-administered FFQ at baseline	7
Drouin-Chartier et al. (9)	USA	NHS, NHS II, HPFS	Both	22–75	32,22, 27	5,903	215,618	Total stroke	Self-administered FFQ at baseline, updated every 4 years	7
Tong et al. (22)	European^a^	EPIC	Both	>20	12.7	7,378	418,329	Total stroke	Interviewer-administered FFQ at baseline	7

a *The study involved 418,329 men and women from 22 centers in nine European countries (Denmark, Germany, Greece, Italy, the Netherlands, Norway, Spain, Sweden, and the UK). UK, United Kingdom; LSS, The Hiroshima/Nagasaki Life Span Study; NIPPON DATA80, National Integrated Project for Prospective Observation of Non-communicable Disease And its Trends in the Aged,1980; NHANES-I, First National Health and Nutrition Examination Survey; PHS, the Physicians' Health Study; NHANES-III The Third National Health and Nutrition Examination Survey; NOMAS, Northern Manhattan Study; CSM, Cohort of Swedish Men; SMC, Swedish Mammography Cohort; GCS, the Golestan Cohort Study; CAPS, Caerphilly prospective cohort study; CKB, the China Kadoorie Biobank collaborative group; GBCS, the Guangzhou Biobank Cohort Study; ARIC, the Atherosclerosis Risk in Communities Study; CARDIA, Coronary Artery Risk Development in Young Adults Study; FHS, Framingham Heart Study; FOS, Framingham Offspring Study; JHS, Jackson Heart Study; MESA, the Multi-Ethnic Study of Atherosclerosis; KIHD, Kuopio Ischaemic Heart Disease Risk Factor Study; NHS, Nurses' Health Study; HPFS, The Health Professional Follow-Up Study; EPIC, the European Prospective Investigation into Cancer and Nutrition. A star rating was used to assess the total quality score for each included study based on the Newcastle Ottawa Scale*.

### Data Analysis

**Figure 2:**
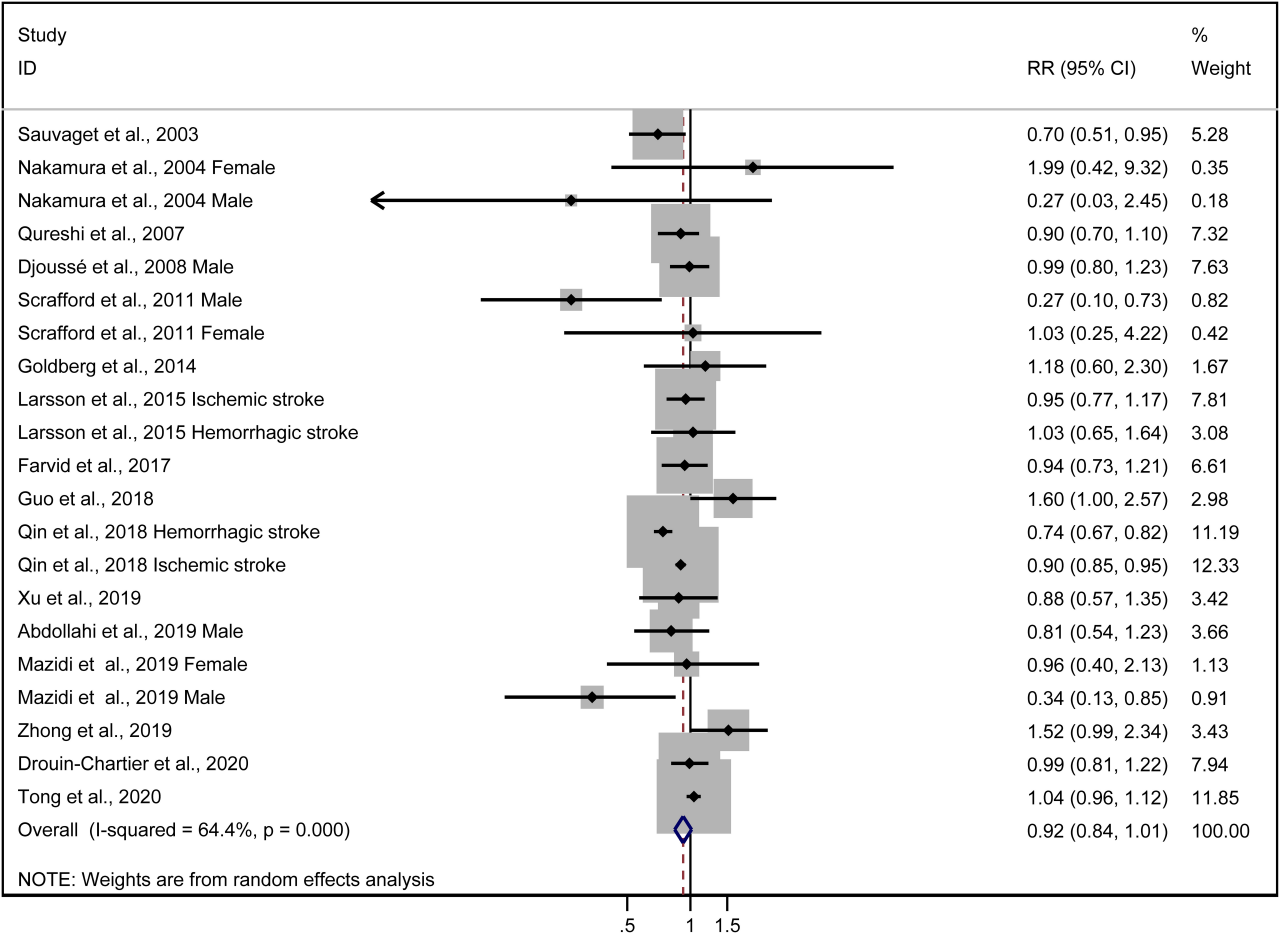
Forest plots showing risk estimates of the association between egg intake and stroke risk.

**Figure 3:**
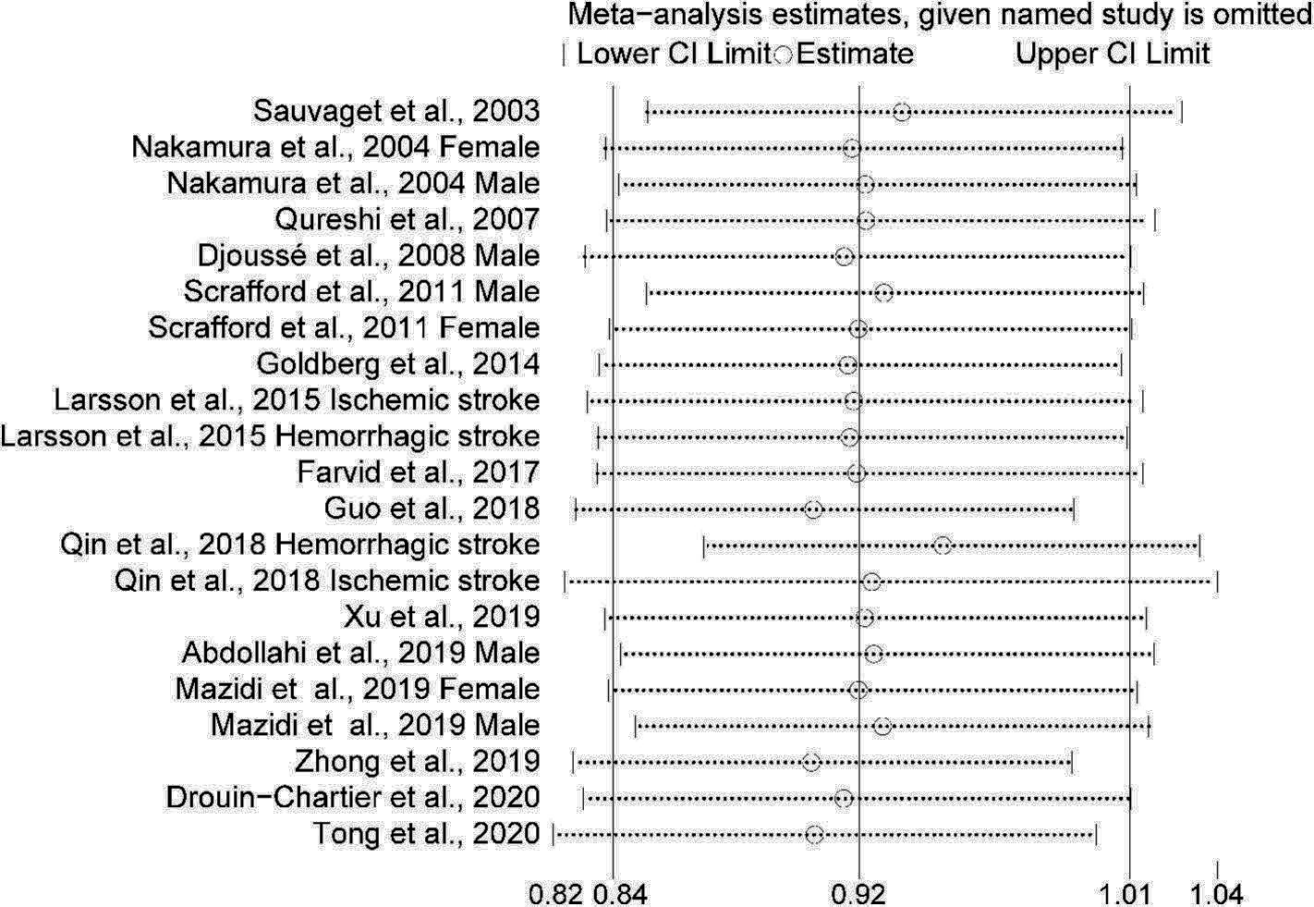
Sensitivity analysis was conducted by removing each study in turn and recalculating the pooled relative risk to determine the impact of each study on the overall risk estimate.

**Figure 4:**
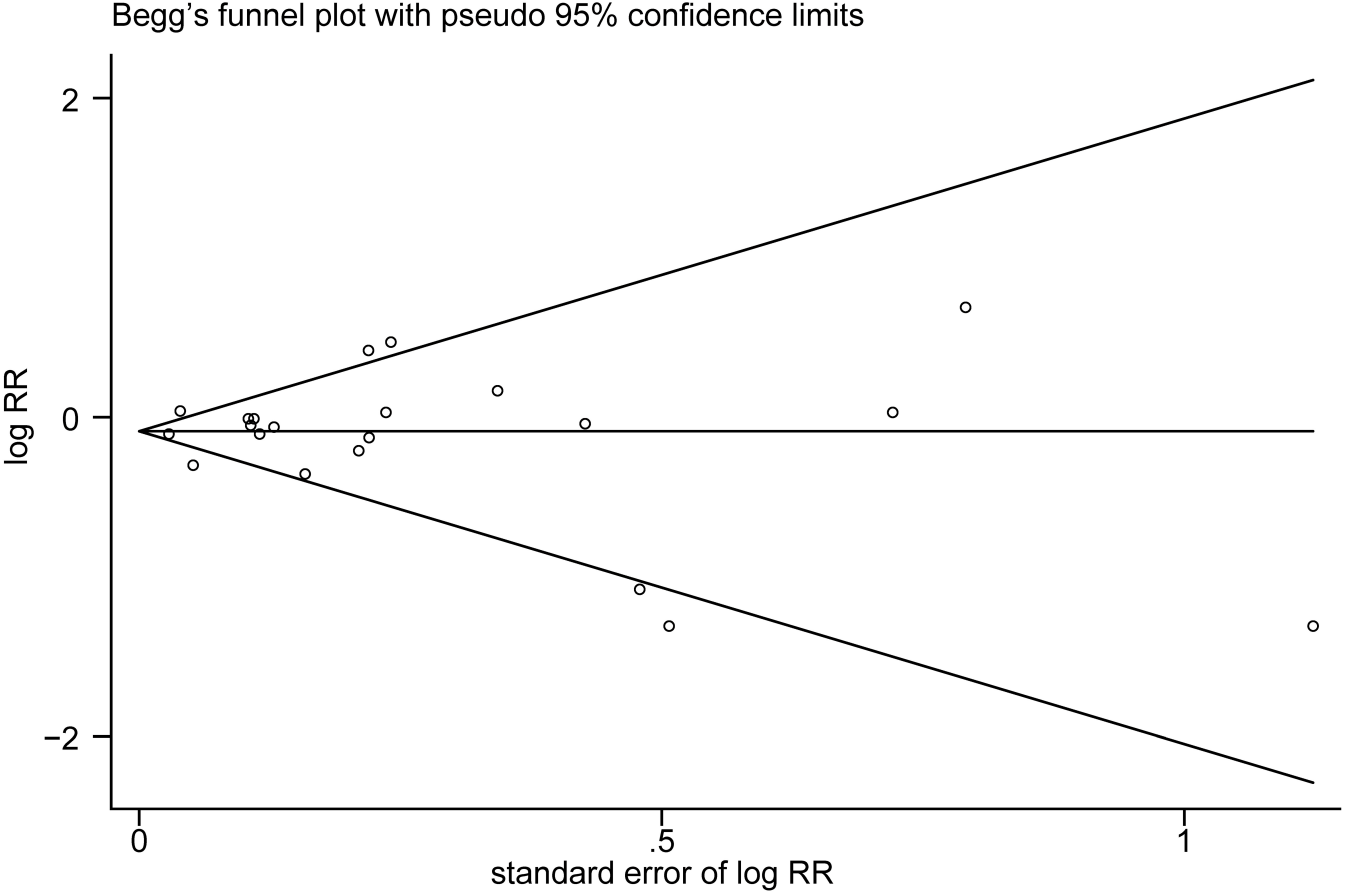
Begg's funnel plot of studies assessing stroke risk with the highest egg intake compared with the lowest egg intake.

The multivariable-adjusted risk estimates of the highest vs. the lowest categories of egg consumption for each study and the combined result are shown in Figure 2. No significant inverse association between egg intake and stroke risk was observed (RR = 0.92, 95% CI: 0.84–1.01). There was moderate heterogeneity across studies (*I*^2^ = 64.5%). Nine of 16 studies reported the relationship between egg intake and stroke mortality (4–6, 8, 15–17, 19, 20). Pooled results indicated that a borderline association was observed (RR = 0.84, 95% CI: 0.71–1.00). Exclusion of one study in turn from the sensitivity analyses showed that a decreased risk of borderline significance was associated with egg intake (Figure 3). Both the Egger linear regression test (*p* = 0.928) and Begg's funnel plots (Figure 4) showed no evidence of publication bias.

The results of subgroup analyses are shown in Table 2. Stratified by geographic region, the inverse association observed was more pronounced in Asia (RR = 0.83, 95% CI: 0.73–0.94), but not in North America (RR = 0.95, 95% CI: 0.77–1.16) or Europe (RR = 1.02, 95% CI: 0.91–1.16). In further subanalyses, we found that a decreased risk of borderline significance was observed for females (RR = 0.88, 95% CI: 0.78–1.00) and ischemic stroke (RR = 0.94, 95% CI: 0.88–1.00), but not for males (RR = 0.89, 95% CI: 0.78–1.01) and hemorrhagic stroke (RR = 0.96, 95% CI: 0.74–1.26). Subgroup analyses by study quality showed no significant difference between the high- and low-quality groups. The pooled RR was 0.93 (0.84–1.03) for the high-quality group and 0.83 (0.84–1.01) for the low-quality group.

**Table 2:** Results of subgroup analysis and heterogeneity test.

**Group**	**No. of studies**	**Pooled RR (95% CI)**	**Heterogeneity**	
			***I*^**2**^**	***P*-value**
All studies	16	0.92(0.84–1.01)	64.5	<0.001
**Geographic region**				
Asia-Pacific	5	0.83(0.73–0.94)	61.1%	0.017
Europe	4	1.02(0.91–1.16)	24.3%	0.259
North America	7	0.95(0.77–1.16)	50.5%	0.040
**Sex**				
Male	9	0.89(0.78–1.01)	55.0%	0.019
Female	6	0.88(0.78–1.00)	30.6%	0.174
**Type of stroke**				
Ischemic stroke	7	0.94(0.88–1.00)	14.9%	0.316
Hemorrhagic stroke	6	0.96(0.74–1.26)	75.2%	0.001
**Study quality**				
High	12	0.93(0.84–1.03)	70.6%	<0.001
Low	4	0.92(0.84–1.01)	41.1%	0.131

**Figure 5:**
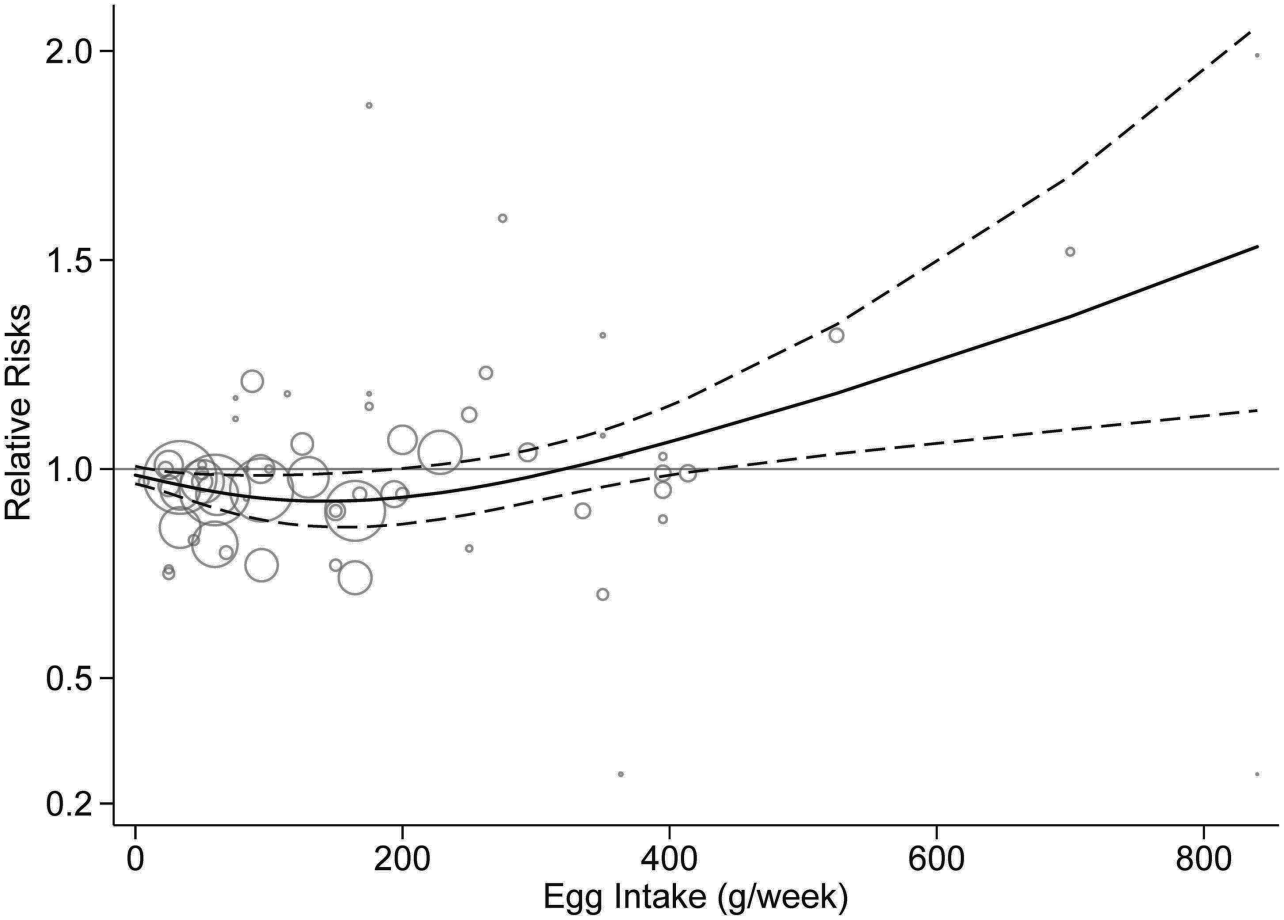
Dose-response analysis of egg intake and stroke risk. The solid line and the dashed line represent the risk estimates and their 95% confidence intervals, respectively.

Fifteen studies were included in the dose-response analysis (4–9, 12, 13, 15–18, 20–22). Based on the results provided in the original studies, we centered the entire reference egg intake to 0 to further assess the potential dose-response effect of egg consumption on stroke risk. Dose-response analysis showed a non-linear association between egg consumption and stroke risk (*p* = 0.02). As shown in Figure 5, egg intake of 50–200 g/week (approximately one to four eggs per week) was associated with a decreased risk, but an increased risk of stroke was associated with increasing egg intake of over 300 g/week (six eggs per week). When egg intake was over 500 g/week, the results were significant.

Based on the NutriGrade scoring system, the quality of meta-evidence was rated as “moderate” (6.8 points), suggesting that further evidence may change the estimate in the future.

## Discussion

To date, egg intake remains a controversial topic as eggs have a multifaceted effect. Twenty-four studies assessed the association between egg consumption and stroke risk, but the results remain inconsistent. In this updated meta-analysis, in a comparison of the highest vs. lowest classifications, no significant association between egg intake and stroke risk was observed. Subgroup analyses showed that sex and stroke type did not exert an influence on this association. However, subgroup analysis by geographic region showed that a significant inverse association was only observed in Asian studies, but not in Western studies. In the USA, frequent egg consumption was associated with unhealthy behaviors such as higher consumption of red meat or processed meat, less intake of skim milk, insufficiency of vegetables or fruits, and lower physical activity (18, 22). Thus, the finding of regional differences suggested that other dietary patterns may attenuate the association between egg consumption and stroke risk, with the exception of possible genetic and environmental factors. In addition, a potential regional difference in the association between stroke and fish and nut consumption was observed in previous meta-analyses (36, 37). Similarly, both meta-analyses which showed a significant inverse association between stroke and fish and nut consumption were found only in Asia. In the dose-response analysis, we found a non-linear association between stroke risk and egg consumption.

Our study may have public health implications. The new 2015 American and 2016 Chinese guidelines removed a limit for dietary cholesterol and recommended eggs as part of a healthy diet (20). Regarding the amount of egg intake, the Chinese guideline recommends healthy adults can consume 40–50 g of egg daily, but no more data were specified in the USA (20). The current study revealed a nearly J-shaped relationship between egg consumption and stroke risk. No significant benefit of increased intake above the intake of four eggs per week was apparent. Furthermore, an increased risk was found in those who consumed more than six eggs per week, although the results did not achieve statistical significance. Therefore, the findings in this study suggest that egg intake should be restricted, but an accurate threshold range should be further explored.

Several possible protective biological mechanisms of egg consumption against stroke have been proposed. First, increased high-density lipoprotein cholesterol (HDL-C) derived from egg phospholipids plays an anti-atherosclerosis role by promoting cholesterol metabolism (38). Second, the ovotransferrin peptide in egg white has a similar antihypertensive effect by preventing vascular smooth muscle remodeling (39). Third, eggs are also rich in lutein and zeaxanthin, which have antioxidant and anti-inflammatory effects (40). Finally, some components in the egg such as vitamins and zinc may have protective effects against stroke (19).

The primary concern related to egg consumption is the adverse effect of high levels of dietary cholesterol in eggs (an egg contains ~175 mg cholesterol) (16). Recent findings on egg intake and the blood lipid profile indicated that egg consumption increased the amount of serum total cholesterol (TC), low-density lipoprotein cholesterol (LDL-C), and HDL-C. Moderate egg consumption (one egg daily) was able to reduce the ratios of TC/HDL-C and LDL-C/HDL-C, but excess egg consumption (more than one egg daily) leads to higher ratios of TC/HDL-C and LDL-C/HDL-C (41). The ratios of TC/HDL-C and LDL-C/HDL-C reflect the balance between the promoting and demoting effects in atherosclerosis, which is an important indicator of stroke risk. A high TC/HDL-C or LDL-C/HDL-C ratio is associated with increased risk of stroke (38). Therefore, it is conceivable that egg has a multifaceted effect.

Limited data on egg intake pattern were reported in the original studies. Most studies reported whole-egg intake or eggs consumed as components of recipes (6, 8, 9, 12, 13, 16, 19, 21, 22), including fried, boiled, poached, deviled, or egg salad, but baked dishes (such as custards and puddings) were not included (6, 19). Other studies did not report the egg intake pattern (4, 5, 7, 15, 17, 18, 20). Moreover, no data regarding the effect of cooking method (e.g., boiled, fried, or others) on the association between egg consumption and stroke risk were reported. Previous findings showed that increased intake of fried fish may be associated with higher amounts of salt, fatty acids, and lipid oxidation products, which are considered risk factors for cardiovascular disease and stroke (36). The nutrient composition of eggs may also be altered depending on the cooking method (20). Thus, this issue requires further verification in future studies.

The relationship between egg consumption and stroke risk has been addressed in several meta-analyses and systematic reviews (19, 20, 22–25). Two meta-analyses published before June 2012, which incorporated half a dozen studies, showed a non-statistically significant inverse association between the highest vs. lowest egg intakes and stroke risk (22, 23). In 2016, Alexander et al. found that a higher egg intake was associated with decreased stroke risk in a meta-analysis of seven studies (24). This finding was confirmed in a later meta-analysis of nine studies, but the shape of the dose-response curve was unclear (20). Another two meta-analyses published in 2019 showed a non-statistically significant positive association (19) or no association between egg intake and stroke risk and no evidence of a non-linear dose-response association (25). Compared with previous meta-analyses, our current study included 16 articles, involving 24 cohort studies, and thus provided relatively reliable estimates. Also, the uniform criterion for egg consumption was defined, and a relatively new field to investigate the possible effects of regional difference and dose-response was provided.

Four major limitations of our study should be acknowledged. First, due to the nature of observational studies, residual confounding factors and measurement errors generated from the original research could mask the true association. For example, data on egg consumption habits were collected using a food questionnaire at baseline, and most of the included studies did not consider the changes in egg consumption during the follow-up years. Second, in a comparison of the highest vs. lowest classifications, some heterogeneity was observed. One potential explanation for this may be associated with different study population characteristics. An alternative explanation is that the highest and reference levels varied markedly. Third, because the included data were from East Asia, Northern America, and Europe, future research in other geographic regions is needed to confirm this finding. Finally, potential publication bias may distort the true association; however, no evidence for publication bias was observed.

In conclusion, our interpretation of the evidence on egg consumption and the risk of stroke is that egg may be part of a healthy diet, but the amount of egg intake should be limited.

## Data Availability Statement

All datasets generated for this study are included in the article/Supplementary Material.

## Author Contributions

HT designed the research and wrote the initial draft. HT, YC, and XY analyzed and synthesized the study data. YZ managed and coordinated the responsibility for the research. All authors contributed to the article and approved the submitted version.

## Conflict of Interest

The authors declare that the research was conducted in the absence of any commercial or financial relationships that could be construed as a potential conflict of interest.
