# Corrigendum: Egg Consumption and Stroke Risk: A Systematic Review and Dose-Response Meta-Analysis of Prospective Studies

**DOI:** 10.3389/fnut.2020.618715

**Published:** 2020-11-23

**Authors:** Hui Tang, Yi Cao, Xiang Yang, Yuekang Zhang

**Affiliations:** ^1^Department of Neurosurgery, West China Hospital, Sichuan University, Chengdu, China; ^2^Department of Neurosurgery, Nanchong Central Hospital, The Second Clinical Medical College, North Sichuan Medical College, Nanchong, China

**Keywords:** egg consumption, stroke risk, systematic review, meta-analysis, dose-response

In the original article, there was a mislabeled Figure 3 as published. **The thirteenth and fourteenth labels in the leftmost column of Figure 3 should have read “Qin et al., 2018 Hemorrhagic stroke” and “Qin et al., 2018 Ischemic stroke”**. The corrected Figure 3 appears below.

**Figure 3:**
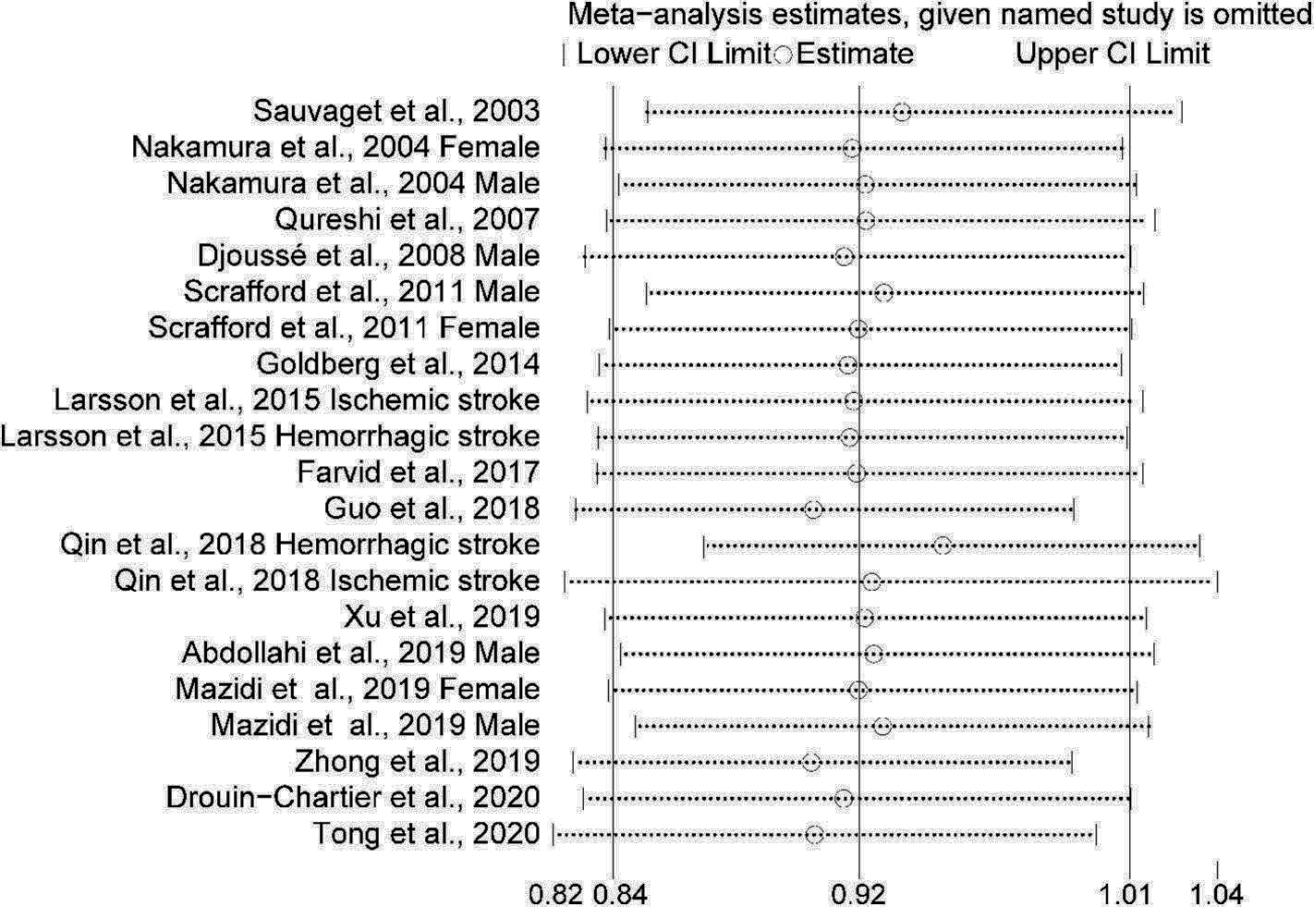
Sensitivity analysis was conducted by removing each study in turn and recalculating the pooled relative risk to determine the impact of each study on the overall risk estimate.

The authors apologize for this error and state that this does not change the scientific conclusions of the article in any way. The original article has been updated.

