# Supplementary material for: Egg Consumption and Stroke Risk: A Systematic Review and Dose-Response Meta-Analysis of Prospective Studies
Source: Front Nutr. 2020 Sep 8;7:153. doi: 10.3389/fnut.2020.00153 (PMC7506150; doi:10.3389/fnut.2020.00153)
Supplement: Supplementary file 1 [file Data_Sheet_1.docx]

SUPPLEMENTAL MATERIAL

**Supplementary Table 1 Database search.**

| **PubMed** | | | |  | **Embase** | | | |  | **Cochrane** | | | |
| --- | --- | --- | --- | --- | --- | --- | --- | --- | --- | --- | --- | --- | --- |
| Search | Query | Items found | Date |  | Search | Query | Items found | Date |  | Search |  | Items found | Date |
| #1 | Search stroke[Title/Abstract] | 242,112 | 2020.06.27 |  | #1 | Search stroke:ab,ti | 383,048 | 2020.06.27 |  | #1 | Search (stroke):ti,ab,kw | 54,082 | 2020.07.01 |
| #2 | Search cerebrovascular[Title/Abstract] | 58,028 | 2020.06.27 |  | #2 | Search cerebrovascular:ab,ti | 80,734 | 2020.06.27 |  | #2 | Search (cerebrovascular):ti,ab,kw | 19,373 | 2020.07.01 |
| #3 | Search #1 OR #2 | 284,843 | 2020.06.27 |  | #3 | Search #1 OR #2 | 441,748 | 2020.06.27 |  | #3 | Search #1 OR #2 | 61,184 | 2020.07.01 |
| #4 | Search egg[Title/Abstract] | 79,565 | 2020.06.27 |  | #4 | Search egg:ab,ti | 95,276 | 2020.06.27 |  | #4 | Search (egg):ti,ab,kw | 3,367 | 2020.07.01 |
| #5 | Search dietary[Title/Abstract] | 252,573 | 2020.06.27 |  | #5 | Search dietary:ab,ti | 317,363 | 2020.06.27 |  | #5 | Search (dietary):ti,ab,kw | 50,261 | 2020.07.01 |
| #6 | Search diet[Title/Abstract] | 329,484 | 2020.06.27 |  | #6 | Search diet:ab,ti | 449,684 | 2020.06.27 |  | #6 | Search (diet):ti,ab,kw | 61,784 | 2020.07.01 |
| #7 | Search #4 OR #5 #6 | 553,208 | 2020.06.27 |  | #7 | Search #4 OR #5 #6 | 728,679 | 2020.06.27 |  | #7 | Search #4 OR #5 #6 | 87,793 | 2020.07.01 |
| #8 | Search prospective[Title/Abstract] | 568,600 | 2020.06.27 |  | #8 | Search prospective:ab,ti | 854,851 | 2020.06.27 |  | #8 | Search (prospective):ti,ab,kw | 221,093 | 2020.07.01 |
| #9 | Search cohort[Title/Abstract] | 535,716 | 2020.06.27 |  | #9 | Search cohort:ab,ti | 906,707 | 2020.06.27 |  | #9 | Search (cohort):ti,ab,kw | 53,307 | 2020.07.01 |
| #10 | Search follow-up[Title/Abstract] | 960,632 | 2020.06.27 |  | #10 | Search 'follow-up':ab,ti | 1,529,936 | 2020.06.27 |  | #10 | Search (follow-up):ti,ab,kw | 243,204 | 2020.07.01 |
| #11 | Search #8 AND #9 AND #10 | 1,755,241 | 2020.06.27 |  | #11 | Search #8 AND #9 AND #10 | 2,767,042 | 2020.06.27 |  | #11 | Search #8 AND #9 AND #10 | 434,750 | 2020.07.01 |
| #12 | Search #3 AND #7 AND #11 | 1,421 | 2020.06.27 |  | #12 | Search #3 AND #7 AND #11 | 2,250 | 2020.06.27 |  | #12 | Search #3 AND #7 AND #11 | 720 | 2020.07.01 |

**Supplementary Table 2 Reasons for study exclusion.**

**(1).Conference abstract without available original data(N=11).**

1. Yaemsiri S, Sen S, Tinker L, Rosamond W, Wassertheil-Smoller S, He K. Dietary fat intake and incidence of ischemic stroke in postmenopausal US women: The womens health initiative. Stroke 2010;41: e223.

2. Park SY, Wilkens LR, Henderson BE, Kolonel LN. Dietary patterns and mortality risk in the multiethnic cohort study. Annals of Nutrition and Metabolism 2013;63: 468.

3. Diaz KM, Booth JN, Calhoun DA, Irvin MR, Howard G, Safford MM, et al. Association of healthy lifestyle factors with all-cause mortality and incident cardiovascular events among individuals with apparent treatment resistant hypertension: The reasons for geographic and racial differences in stroke (REGARDS) study. Circulation 2014;129.

4. Pearson KE, Voeks JH, Carson AP, Shikany JS, Safford MM, Fernandez JR, et al. Associations of dietary patterns and cardiovascular disease risk do not differ by diabetes status. Circulation 2014;129.

5. Guo J, Lovegrove JA, Cockcroft JR, Elwood PC, Pickering JE, Givens DI. Egg consumption and cardiovascular disease events-evidence from the Caerphilly prospective cohort study. Proceedings of the Nutrition Society 2015;74.

6. Spence JD, Judd S, Howard V, Safford M, Howard G. Effect of dietary cholesterol and egg consumption on mortality and cardiovascular risk in the regards study. Stroke 2015;46.

7. Qi L, Lv J, Yu C, Guo Y, Bian Z, Yang L, et al. Adherence to healthy lifestyle and cardiovascular diseases in chinese. Circulation 2016;134.

8. Nakamura Y, Okamura T, Kita Y, Miura K, Okayama A, Ueshima H. Re-evaluation of the relations of egg consumption to serum total cholesterol, cause-specific and all-cause mortality. Circulation 2017;135.

9. Zhang J, Du W, Huang F, Su C, Zhang J, Jiang H, et al. Egg consumption and risk of cardiovascular diseases, evidence from China health and nutrition survey 2000-2011. FASEB Journal 2018;32.

10. Zhong VW, Van Horn L, Cornelis MC, Wilkins JT, Ning H, Carnethon MR, et al. Associations of dietary cholesterol or egg consumption with incident cardiovascular disease and mortality: The lifetime risk pooling project. Circulation 2018;138.

11. Rees K, Takeda A, Martin N, Ellis L, Wijesekara D, Vepa A, et al. Mediterranean-style diet for the primary and secondary prevention of cardiovascular disease. Cochrane Database of Systematic Reviews 2019;2019.

**(2) Reporting data about dietary patterns or no available data about stroke and egg (N=69).**

1. Kagan A, Popper JS, Rhoads GG, Yano K. Dietary and other risk factors for stroke in Hawaiian Japanese men. Stroke 1985;16: 390-396.

2. McGee D, Reed D, Stemmerman G, Rhoads G, Yano K, Feinleib M. The relationship of dietary fat and cholesterol to mortality in 10 years: the Honolulu Heart Program. Int J Epidemiol 1985;14: 97-105.

3. Lapidus L, Andersson H, Bengtsson C, Bosaeus I. Dietary habits in relation to incidence of cardiovascular disease and death in women: A 12-year follow-up of participants in the population study of women in Gothenburg, Sweden. American Journal of Clinical Nutrition 1986;44: 444-448.

4. Fukuzawa Y, Kishimoto T, Abe M, Tada M, Masuda N, Shigematsu T. [Influence of the changes in food intake patterns and smoking and drinking habits on stroke--20-year follow-up survey in the Oki-Islands, Shimane Prefecture, Japan]. Nihon eiseigaku zasshi Japanese journal of hygiene 1990;45: 890-903.

5. Key TJ, Thorogood M, Appleby PN, Burr ML. Dietary habits and mortality in 11,000 vegetarians and health conscious people: results of a 17 year follow up. BMJ (Clinical research ed) 1996;313: 775-779.

6. Ross RK, Yuan JM, Henderson BE, Park J, Gao YT, Yu MC. Prospective evaluation of dietary and other predictors of fatal stroke in Shanghai, China. Circulation 1997;96: 50-55.

7. Iso H, Stampfer MJ, Manson JE, Rexrode K, Hu FB, Hennekens CH, et al. Prospective study of fat and protein intake and risk of intraparenchymal hemorrhage in women. Circulation 2001;103: 856-863.

8. Iso H, Sato S, Kitamura A, Naito Y, Shimamoto T, Komachi Y. Fat and protein intakes and risk of intraparenchymal hemorrhage among middle-aged Japanese. American Journal of Epidemiology 2003;157: 32-39.

9. Fung TT, Stampfer MJ, Manson JE, Rexrode KM, Willett WC, Hu FB. Prospective study of major dietary patterns and stroke risk in women. Stroke 2004;35: 2014-2019.

10. Hobbs R. Risk of stroke in men is not associated with dietary fat intake. Evidence-Based Healthcare 2004;8: 74-76.

11. Cai H, Shu XO, Gao YT, Li H, Yang G, Zheng W. A prospective study of dietary patterns and mortality in Chinese women. Epidemiology 2007;18: 393-401.

12. Shimazu T, Kuriyama S, Hozawa A, Ohmori K, Sato Y, Nakaya N, et al. Dietary patterns and cardiovascular disease mortality in Japan: A prospective cohort study. International Journal of Epidemiology 2007;36: 600-609.

13. Chiuve SE, Rexrode KM, Spiegelman D, Logroscino G, Manson JE, Rimm EB. Primary prevention of stroke by healthy lifestyle. Circulation 2008;118: 947-954.

14. Fung TT, Chiuve SE, McCullough ML, Rexrode KM, Logroscino G, Hu FB. Adherence to a DASH-style diet and risk of coronary heart disease and stroke in women. Archives of Internal Medicine 2008;168: 713-720.

15. Heidemann C, Schulze MB, Franco OH, Van Dam RM, Mantzoros CS, Hu FB. Dietary patterns and risk of mortality from cardiovascular disease, cancer, and all causes in a prospective cohort of women. Circulation 2008;118: 230-237.

16. Fung TT, Rexrode KM, Mantzoros CS, Manson JE, Willett WC, Hu FB. Mediterranean diet and incidence of and mortality from coronary heart disease and stroke in women. Circulation 2009;119: 1093-1100.

17. Panagiotakos D, Pitsavos C, Chrysohoou C, Palliou K, Lentzas I, Skoumas I, et al. Dietary patterns and 5-year incidence of cardiovascular disease: A multivariate analysis of the ATTICA study. Nutrition, Metabolism and Cardiovascular Diseases 2009;19: 253-263.

18. Panagiotakos DB, Chrysohoou C, Pitsavos C, Skoumas J, Lentzas J, Masoura D, et al. Mediterranean diet is associated with lower incidence of CVD events: A five-year follow-up of the ATTICA study. Journal of the American College of Cardiology 2009;53: A226.

19. Parikh A, Lipsitz SR, Natarajan S. Association between a DASH-like diet and mortality in adults with hypertension: Findings from a population-based follow-up study. American Journal of Hypertension 2009;22: 409-416.

20. Preis SR, Stampfer MJ, Spiegelman D, Willett WC, Rimm EB. Lack of association between dietary protein intake and risk of stroke among middle-aged men. American Journal of Clinical Nutrition 2010;91: 39-45.

21. Atkinson C, Whitley E, Ness A, Baker I. Associations between types of dietary fat and fish intake and risk of stroke in the Caerphilly Prospective Study (CaPS). Public Health 2011;125: 345-348.

22. Belin RJ, Greenland P, Allison M, Martin L, Shikany JM, Larson J, et al. Diet quality and the risk of cardiovascular disease: The Women's Health Initiative (WHI). American Journal of Clinical Nutrition 2011;94: 49-57.

23. Gardener H, Wright CB, Gu Y, Demmer RT, Boden-Albala B, Elkind MS, et al. Mediterranean-style diet and risk of ischemic stroke, myocardial infarction, and vascular death: the Northern Manhattan Study. Am J Clin Nutr 2011;94: 1458-1464.

24. Hlebowicz J, Persson M, Gullberg B, Sonestedt E, Wallstrom P, Drake I, et al. Food patterns, inflammation markers and incidence of cardiovascular disease: the Malmo Diet and Cancer study. J Intern Med 2011;270: 365-376.

25. Nakamura Y, Kiyohara Y, Okamura T, Higashiyama A, Watanabe M, Kadota A, et al. Saturated fatty acids intake, polyunsaturated fatty acid intake and coronary heart disease mortality in Japan: NIPPON DATA90 1990-2005. European Heart Journal 2011;32: 722.
26. Tada N, Maruyama C, Koba S, Tanaka H, Birou S, Teramoto T, et al. Japanese dietary lifestyle and cardiovascular disease. Journal of Atherosclerosis and Thrombosis 2011;18: 723-734.

27. Hoevenaar-Blom MP, Nooyens AC, Kromhout D, Spijkerman AM, Beulens JW, van der Schouw YT, et al. Mediterranean style diet and 12-year incidence of cardiovascular diseases: the EPIC-NL cohort study. PLoS One 2012;7: e45458.

28. Larsson SC, Virtamo J, Wolk A. Dietary protein intake and risk of stroke in women. Atherosclerosis 2012;224: 247-251.

29. Larsson SC, Virtamo J, Wolk A. Dietary fats and dietary cholesterol and risk of stroke in women. Atherosclerosis 2012;221: 282-286.

30. Lip GY, Rasmussen LH, Skjoth F, Overvad K, Larsen TB. Stroke and mortality in patients with incident heart failure: the Diet, Cancer and Health (DCH) cohort study. BMJ Open 2012;2.

31. Nagata C, Nakamura K, Wada K, Oba S, Tsuji M, Tamai Y, et al. Total fat intake is associated with decreased mortality in Japanese men but not in women. Journal of Nutrition 2012;142: 1713-1719.

32. Chan R, Chan D, Woo J. The association of a priori and a posterior dietary patterns with the risk of incident stroke in chinese older people in Hong Kong. Journal of Nutrition, Health and Aging 2013;17: 866-874.

33. Chen Y, McClintock TR, Segers S, Parvez F, Islam T, Ahmed A, et al. Prospective investigation of major dietary patterns and risk of cardiovascular mortality in Bangladesh. International Journal of Cardiology 2013;167: 1495-1501.

34. Estruch R, Ros E, Salas-Salvadó J, Covas MI, Corella D, Arós F, et al. Primary prevention of cardiovascular disease with a Mediterranean diet. New England Journal of Medicine 2013;368: 1279-1290.

35. Shikany JM, Newby PK, Safford MM, Durant RW, Brown TM, Roth DL, et al. Specific dietary patterns are associated with risk of acute coronary heart disease in the reasons for geographic and racial differences in stroke (regards) study. Circulation 2013;127.

36. Diaz KM, Booth IJN, Calhoun DA, Irvin MR, Howard G, Safford MM, et al. Healthy lifestyle factors and risk of cardiovascular events and mortality in treatment-resistant hypertension: The Reasons for Geographic and Racial Differences in Stroke study. Hypertension 2014;64: 465-471.

37. Larsson SC, Akesson A, Wolk A. Healthy diet and lifestyle and risk of stroke in a prospective cohort of women. Neurology 2014;83: 1699-1704.

38. Larsson SC, Akesson A, Wolk A. Overall diet quality and risk of stroke: a prospective cohort study in women. Atherosclerosis 2014;233: 27-29.

39. Struijk EA, May AM, Wezenbeek NL, Fransen HP, Soedamah-Muthu SS, Geelen A, et al. Adherence to dietary guidelines and cardiovascular disease risk in the EPIC-NL cohort. Int J Cardiol 2014;176: 354-359.

40. Biesbroek S, van der AD, Brosens MC, Beulens JW, Verschuren WM, van der Schouw YT, et al. Identifying cardiovascular risk factor-related dietary patterns with reduced rank regression and random forest in the EPIC-NL cohort. Am J Clin Nutr 2015;102: 146-154.

41. Georgousopoulou EN, Panagiotakos DB, Pitsavos C, Stefanadis C. Assessment of diet quality improves the classification ability of cardiovascular risk score in predicting future events: The 10-year follow-up of the ATTICA study (2002-2012). European Journal of Preventive Cardiology 2015;22: 1488-1498.

42. Georgousopoulou EN, Pitsavos C, Panagiotakos D, Chrysohoou C, Skoumas I, Chatzigeorgiou M, et al. Adherence to mediterranean is the most important protector against the development of fatal and non-fatal cardiovascular event: 10-year follow-up (2002-12) of the Attica study. Journal of the American College of Cardiology 2015;65: A1449.

43. Guasch-Ferre M, Babio N, Martinez-Gonzalez MA, Corella D, Ros E, Martin-Pelaez S, et al. Dietary fat intake and risk of cardiovascular disease and all-cause mortality in a population at high risk of cardiovascular disease. 2015;102: 1563-1573.

44. Jacques PF, Cassidy A, Rogers G, Peterson JJ, Dwyer JT. Dietary flavonoid intakes and CVD incidence in the Framingham Offspring Cohort. The British journal of nutrition 2015;114: 1496-1503.

45. Larsson SC, Akesson A, Wolk A. Primary prevention of stroke by a healthy lifestyle in a high-risk group. Neurology 2015;84: 2224-2228.

46. Mattiello A, Chiodini P, Santucci de Magistris M, Krogh V, Grioni S, Fasanelli F, et al. [Dietary habits and cardiovascular disease: the experience of EPIC Italian collaboration]. Epidemiologia e prevenzione 2015;39: 339-344.

47. Stefler D, Pikhart H, Kubinova R, Pajak A, Malyutina S, Peasey A, et al. Mediterranean diet and mortality in eastern Europeans: Results from the HAPIEE study. European Journal of Epidemiology 2015;30: 725-726.

48. Tektonidis TG, Akesson A, Gigante B, Wolk A, Larsson SC. A Mediterranean diet and risk of myocardial infarction, heart failure and stroke: A population-based cohort study. Atherosclerosis 2015;243: 93-98.

49. Stewart RAH, Wallentin L, Benatar J, Danchin N, Hagström E, Held C, et al. Dietary patterns and the risk of major adverse cardiovascular events in a global study of high-risk patients with stable coronary heart disease. European Heart Journal 2016;37: 1993-2001.

50. Van Lee L, Geelen A, Kiefte-De Jong JC, Witteman JCM, Hofman A, Vonk N, et al. Adherence to the Dutch dietary guidelines is inversely associated with 20-year mortality in a large prospective cohort study. European Journal of Clinical Nutrition 2016;70: 262-268.

51. Galbete Ciaurriz C, Kröger J, Jannasch F, Schwingshackl L, Schwedhelm C, Iqbal K, et al. Nordic diet, mediterranean diet, and the risk of chronic diseases: The epic-potsdam study. Annals of Nutrition and Metabolism 2017;71: 320.

52. Hansen CP, Overvad K, Kyrø C, Olsen A, Tjønneland A, Johnsen Sø P, et al. Adherence to a Healthy Nordic Diet and Risk of Stroke: A Danish Cohort Study. Stroke 2017;48: 259-264.

53. Hirahatake KM, Odegaard A, Wong N, Malik S, Jiang L, Shikany J, et al. Diet quality and cardiovascular disease risk in postmenopausal women with type 2 diabetes: The women's health initiative. Circulation 2017;135.

54. Mertens E, Markey O, Geleijnse JM, Givens DI, Lovegrove JA. Dietary patterns in relation to cardiovascular disease incidence and risk markers in a middle-aged british male population: Data from the caerphilly prospective study. Nutrients 2017;9.

55. Micha R, Penalvo JL, Cudhea F, Imamura F, Rehm CD, Mozaffarian D. Association Between Dietary Factors and Mortality From Heart Disease, Stroke, and Type 2 Diabetes in the United States. Jama 2017;317: 912-924.

56. Mohammadifard N, Talaei M, Sadeghi M, Oveisegharan S, Golshahi J, Esmaillzadeh A, et al. Dietary patterns and mortality from cardiovascular disease: Isfahan Cohort Study. European Journal of Clinical Nutrition 2017;71: 252-258.

57. Ozawa M, Yoshida D, Hata J, Ohara T, Mukai N, Shibata M, et al. Dietary Protein Intake and Stroke Risk in a General Japanese Population: The Hisayama Study. Stroke 2017;48: 1478-1486.

58. Prentice RL, Aragaki AK, Van Horn L, Thomson CA, Beresford SA, Robinson J, et al. Low-fat dietary pattern and cardiovascular disease: results from the Women's Health Initiative randomized controlled trial. Am J Clin Nutr 2017;106: 35-43.

59. Stefler D, Malyutina S, Kubinova R, Pajak A, Peasey A, Pikhart H, et al. Mediterranean diet score and total and cardiovascular mortality in Eastern Europe: the HAPIEE study. European Journal of Nutrition 2017;56: 421-429.

60. Van Horn L, Ning H, Steffen L, Jacobs D, Shikany J, Miedema M, et al. Dietary factors associated with cardiovascular outcomes: 25 year findings from the coronary artery risk development in young adults (CARDIA) study. Circulation 2017;135.

61. Aigner A, Becher H, Jacobs S, Wilkens LR, Boushey CJ, Le Marchand L, et al. Low diet quality and the risk of stroke mortality: The multiethnic cohort study. European Journal of Clinical Nutrition 2018;72: 1035-1045.

62. Biesbroek S, Kneepkens MC, Van Den Berg SW, Fransen HP, Beulens JW, Peeters PHM, et al. Dietary patterns within educational groups and their association with CHD and stroke in the European Prospective Investigation into Cancer and Nutrition-Netherlands cohort. British Journal of Nutrition 2018;119: 949-956.

63. Czekajlo A, Rozanska D, Zatonska K, Szuba A, Regulska-Ilow B. Association between dietary patterns and cardiovascular disease risk in selected population of Polish adults. European Heart Journal 2018;39: 293.

64. Dahm CC, Johnsen SP, Schmidt EB, Overvad K, Jakobsen MU, Jones NRV. Accordance to the Dietary Approaches to Stop Hypertension diet pattern and cardiovascular disease in a British, population-based cohort. Eur J Nutr 2018;33: 235-244.

65. Galbete C, Kröger J, Jannasch F, Iqbal K, Schwingshackl L, Schwedhelm C, et al. Nordic diet, Mediterranean diet, and the risk of chronic diseases: The EPIC-Potsdam study. BMC Medicine 2018;16.

66. Mertens E, Markey O, Geleijnse JM, Lovegrove JA, Givens DI. Adherence to a healthy diet in relation to cardiovascular incidence and risk markers: evidence from the Caerphilly Prospective Study. European Journal of Nutrition 2018;57: 1245-1258.

67. Paterson KE, Myint PK, Jennings A, Bain LKM, Lentjes MAH, Khaw KT, et al. Mediterranean Diet Reduces Risk of Incident Stroke in a Population With Varying Cardiovascular Disease Risk Profiles. Stroke 2018: 2415-2420.

68. Kobayashi M, Sasazuki S, Shimazu T. Association of dietary diversity with total mortality and major causes of mortality in the Japanese population: JPHC study. 2019.

69. Yamagishi K, Iso H, Kokubo Y, Saito I, Yatsuya H, Ishihara J, et al. Dietary intake of saturated fatty acids and incident stroke and coronary heart disease in Japanese communities: The JPHC Study. European Heart Journal 2013;34: 1225-1232.

**(3). Shorter follow-up than others on the same cohort or duplicated data (N=3).**

1. Hu FB, Stampfer MJ, Rimm EB, Manson JE, Ascherio A, Colditz GA, et al. A prospective study of egg consumption and risk of cardiovascular disease in men and women. JAMA, 1999;281(15):1387-94.

2. Haring B, Misialek JR, Rebholz CM, et al. Association of Dietary Protein Consumption With Incident Silent Cerebral Infarcts and Stroke: The Atherosclerosis Risk in Communities (ARIC) Study. Stroke. 2015;46(12):3443-50.

3. Bernstein AM, Pan A, Rexrode KM, Stampfer M, Hu FB, Mozaffarian D, Willett WC. Dietary protein sources and the risk of stroke in men and women. Stroke. 2012;43(3):637-44.

**(4). Involved total CVD (i.e. no data reported separately for stroke) (N=3).**

1. Díez-Espino J, Basterra-Gortari FJ, Salas-Salvadó J, et al. Egg consumption and cardiovascular disease according to diabetic status: The PREDIMED study. Clin Nutr. 2017;36(4):1015-1021.

2. Zazpe I, Beunza JJ, Bes-Rastrollo M, et al. Egg consumption and risk of cardiovascular disease in the SUN Project. Eur J Clin Nutr. 2011;65(6):676-82.

3. Houston DK, Ding J, Lee JS, et al. Dietary fat and cholesterol and risk of cardiovascular disease in older adults: the Health ABC Study. Nutr Metab Cardiovasc Dis. 2011;21(6):430-7

**(5). No accurate data about the reference category (N=3).**

1.Yaemsiri S, Sen S, Tinker L, Rosamond W, Wassertheil-Smoller S, He K. Trans fat, aspirin, and ischemic stroke in postmenopausal women. Ann Neurol. 2012;72(5):704-15.

2. Misirli G, Benetou V, Lagiou P, Bamia C, Trichopoulos D, Trichopoulou A. Relation of the traditional Mediterranean diet to cerebrovascular disease in a Mediterranean population. Am J Epidemiol. 2012;176(12):1185-92.

3.Wang, JB, Fan JH, Dawsey SM, Sinha R, Freedman ND, Taylor PR, Qiao YL, et al. Dietary components and risk of total, cancer and cardiovascular disease mortality in the Linxian Nutrition Intervention Trials cohort in China. Sci Rep. 2016;6:22619.

**Supplementary Table 3 Main characteristic of included studies.**

| **References** | **Details** | **Exposure categories** | **RR (95% CI)** | **Adjusted variables** | **Egg intake pattern** |
| --- | --- | --- | --- | --- | --- |
| Sauvaget et al. 2003[4] |  | None | 1.00 (reference) | Age, sex, birth cohort, smoking history, alcohol, BMI, education, histories of diabetes, histories of hypertension, radiation dose, and city | Not Report |
|  |  | ≤1 time/week | 0.75(0.55-1.01) |  |  |
|  |  | 2-4 times/week | 0.77(0.57-1.03) |  |  |
|  |  | Almost daily | 0.70(0.51- 0.95) |  |  |
| Nakamura et al. 2004 [5] | Female | 1/d | 1.00 (reference) | Age, serum creatinine, total cholesterol, blood glucose, BMI, systolic and diastolic blood pressures, use of blood pressure–lowering drugs, smoking history, and alcohol intake. | Not Report |
|  |  | Seldom | 0.78(0.35-1.73) |  |  |
|  |  | 1–2/week | 0.79(0.47-1.33) |  |  |
|  |  | 1/2 d | 1.46(0.89-2.40) |  |  |
|  |  | ≥2/d | 1.22(0.29-5.17) |  |  |
|  | Male | 1/d | 1.00 (reference) |  |  |
|  |  | Seldom | 0.93(0.36-2.40) |  |  |
|  |  | 1–2/week | 1.09(0.69-1.72) |  |  |
|  |  | 1/2 day | 1.10(0.68-1.76) |  |  |
|  |  | ≥2/day | 0.25(0.03-1.81) |  |  |
| Qureshi et al. 2007 [6] |  | <1 /week | 1.00 (reference) | Age, gender, race/ethnicity, systolic blood pressure, diabetes mellitus, serum cholesterol, smoking history, BMI, and educational status. | Fried, boiled, poached, deviled, or egg salad. Eggs in cooked or baked dishes (as custards, puddings) were not included. |
|  |  | 1-6/week | 0.9(0.7-1.0) |  |  |
|  |  | >6 week | 0.9(0.7-1.1) |  |  |
| Djoussé et al. 2008 [7] | Male | <1 /week | 1.00 (reference) | Age, BMI, smoking history, history of hypertension, vitamin intake, alcohol consumption, vegetable consumption, breakfast cereal, physical activity, treatment arm atrial fibrillation, diabetes mellitus, hypercholesterolemia, and parental history of premature myocardial infarction. | Not Report |
|  |  | 1/week | 0.96 (0.82-1.13) |  |  |
|  |  | 2-4/week | 1.06 (0.91-1.24) |  |  |
|  |  | 5-6/week | 1.13 (0.89-1.42) |  |  |
|  |  | 7+/week | 0.99 (0.80-1.23) |  |  |
| Scrafford et al. 2011 [8] | Male | 0.27/wk | 1.00 (reference) | Age, energy, marital status, educational status, race/ethnicity, smoking history, BMI, WHR, diabetes, hypertension, and dietary variables | Scrambled, fried, omelettes, hard-boiled and egg salad |
|  |  | 1.93/week | 1.00(0.49-2.02) |  |  |
|  |  | 7.54/week | 0.27(0.10-0.73) |  |  |
|  | Female | 0.24/week | 1.00 (reference) |  |  |
|  |  | 1.79/week | 0.93 (0.46-1.90) |  |  |
|  |  | 7.41/week | 1.03(0.25-4.22) |  |  |
| Goldberg et al. 2014 [12] |  | <1/month | 1.00 (reference) | Age, sex, race/ethnicity, BMI, diabetes, hypertension, LDL, HDL, TG, cholesterol-lowering medication, moderate alcohol use, moderate-heavy physical activity, smoking history, high-school completion, family history of stroke in siblings, family history of MI in siblings, daily consumption of saturated fat, unsaturated fat, carbohydrates, and protein. | Whole eggs |
|  |  | 1/month | 0.97(0.69-1.37) |  |  |
|  |  | 2-3/month | 0.76(0.50-1.14) |  |  |
|  |  | 1/week | 0.83(0.60-1.16) |  |  |
|  |  | ≥2/week | 1.18(0.60-2.30) |  |  |
| Larsson et al. 2015 [13] | IS | <3/month | 1.00 (reference) | Age, education, family history of myocardial infarction before 60 y of age; smoking history, aspirin use; walking/bicycling; exercise; BMI; history of hypertension, hypercholesterolemia, diabetes, and intakes of total energy, alcohol, fruit and vegetables, and processed meat. | Eggs/omelets |
|  |  | 1–2/week | 0.97 (0.90-1.05) |  |  |
|  |  | 3–6/week | 1.07(0.95,-1.20) |  |  |
|  |  | ≥1/day | 0.95(0.77-1.17) |  |  |
|  | HS | <3/month | 1.00 (reference) |  |  |
|  |  | 1–2/week | 0.97(0.82-1.14) |  |  |
|  |  | 3–6/week | 0.94(0.72-1.22) |  |  |
|  |  | ≥1/day | 1.03(0.65-1.64) |  |  |
| Farvid et al., 2017 [15] |  | 0/day | 1.00 (reference) | Age, gender, ethnicity, education, marital status, residency, smoking history, opium use, alcohol, BMI, systolic blood pressure, occupational physical activity, family history of cancer, wealth score, medication, and energy intake. | Not Report |
|  |  | 0.06 serving/day | 1.00(0.79-1.26) |  |  |
|  |  | 0.18 serving/day | 0.80(0.62-1.04) |  |  |
|  |  | 0.48 serving/day | 0.94(0.73-1.21) |  |  |
| Guo et al., 2018 [16] |  | ≤1/week | 1.00 (reference) | Age, BMI, total energy intake, alcohol consumption, smoking history, energy expenditure, social class), family history of myocardial infarction, diabetes mellitus, sugar intake, fruit consumption, red meat consumption, and fiber. | Whole eggs and dishes such as omelettes and scrambled eggs |
|  |  | 1-2/week | 1.01(0.65-1.56) |  |  |
|  |  | 2-3/week | 1.00(0.64-1.55) |  |  |
|  |  | 3-5/week | 1.15(0.72-1.84) |  |  |
|  |  | ≥5/week | 1.60 (1.00-2.57) |  |  |
| Qin et al. 2018 [17] | HS | 0.29/day | 1.00 (reference) | Age, sex, education level, household income, marital status, alcohol consumption, smoking history, physical activity, BMI, WHR, prevalent hypertension, use of aspirin, family history of cardiovascular disease, intake of multivitamin supplementation, and dietary pattern. | Not Report |
|  |  | 0.36/day | 0.86 (0.79-0.93) |  |  |
|  |  | 0.46/day | 0.82 (0.76-0.88) |  |  |
|  |  | 0.56/day | 0.77(0.70-0.86) |  |  |
|  |  | 0.76/day | 0.74(0.67-0.82) |  |  |
|  | IS | 0.29/day | 1.00 (reference) |  |  |
|  |  | 0.36/day | 0.98 (0.94-1.03 |  |  |
|  |  | 0.46/day | 0.95 (0.91-1.00) |  |  |
|  |  | 0.56/day | 0.95 (0.9-1.00) |  |  |
|  |  | 0.76/day | 0.90(0.85-0.95) |  |  |
| Xu et al. 2019 [20] |  | <1 /week | 1.00 (reference) | Age, sex, education, occupation, family income, smoking history, physical activity, alcohol drinking, self-rated health, and chronic disease history (diabetes, hypertension and dyslipidemia) | Not Report |
|  |  | 1-2 /week | 0.99(0.75-1.3) |  |  |
|  |  | 3-4/week | 0.90(0.68-1.20) |  |  |
|  |  | 5-6/week | 0.81(0.47-1.38) |  |  |
|  |  | 7+/week | 0.88(0.57-1.35) |  |  |
| Mazidi et al. 2019 [19] | Female | Q1 | 1.00 (reference) | Age, race, education, marital status, poverty to income ratio, total energy intake, physical activity, smoking history, alcohol consumption, intake of fiber and meat, BMI, hypertension, and diabetes. | Whole eggs (eggs used in baking were not included) |
|  |  | Q2 | 0.98(0.52-2.11) |  |  |
|  |  | Q3 | 0.96(0.40-2.13) |  |  |
|  | Male | Q1 | 1.00(reference) |  |  |
|  |  | Q2 | 0.28(0.10-0.75) |  |  |
|  |  | Q3 | 0.34(0.13-0.85) |  |  |
| Zhong et al. 2019 [18] |  | 0/day | 1.00 (reference) | Age, sex, race/ethnicity, education, total energy, smoking history, cohort-specific physical activity z-score, alcohol consumption, use of hormone replacement therapy, BMI, diabetes status, systolic blood pressure, use of anti-hypertensive medications, HDL-C, non-HDL-C, and use of lipid-lowering medications. | Exclude egg substitute |
|  |  | <0.5/day | 1.21 (1.04-1.42) |  |  |
|  |  | 0.5-1/day | 1.23 (0.94-1.62) |  |  |
|  |  | 1-2/ day | 1.32 (1.03-1.68) |  |  |
|  |  | ≥2/day | 1.52 (0.99-2.34) |  |  |
| Abdollahi et al. 2019 [21] |  | <15 g/d | 1.00 (reference) | Age, examination year, energy intake, BMI, smoking history, physical activity, hypertension medication, intakes of alcohol, fruit, berries, and vegetables. | Whole eggs (including the intake of eggs in mixed dishes and recipes) |
|  |  | 15-26 g/d | 1.01 (0.69-1.47) |  |  |
|  |  | 27-45 g/d | 1.10 (0.76-1.61) |  |  |
|  |  | >45 g/d | 0.81 (0.54-1.23) |  |  |
| Drouin-Chartier et al. 2020 [9] |  | <1/month | 1.00 (reference) | Age, stratified by calendar time, cohort, race, family history of myocardial infarction, baseline hypercholesterolemia, baseline hypertension, smoking status, body mass index, physical activity, oral contraceptive use, postmenopausal hormone use, alcohol intake, multivitamin use, updated cumulative average of daily intake of total calories, full-fat milk, bacon, other processed meats, refined grains, fruits, vegetables), potatoes, coffee, fruit juices, and sugar-sweetened beverages. | Whole eggs and egg products |
|  |  | 1-4/month | 1.00 (0.89-1.13) |  |  |
|  |  | 1-3/week | 1.00 (0.89-1.13) |  |  |
|  |  | 3-5/week | 0.94 (0.82-1.06) |  |  |
|  |  | 5-<7/week | 1.04 (0.86-1.25) |  |  |
|  |  | ≥1/day | 0.99 (0.81-1.22) |  |  |
| Tong et al. 2020 [22] |  | 3.0g/day median | 1.00 (reference) | Age, smoking history, history of diabetes, prior hypertension, prior hyperlipidaemia, Cambridge physical activity index, employment status, level of education completed, current alcohol consumption, body mass index), and observed intake of energy, and stratified by sex and EPIC center. | Whole eggs and egg products, such as eggs consumed as components of recipes |
|  |  | 7.8g/day median | 0.95 (0.87-1.03) |  |  |
|  |  | 13.7g/day median | 0.94 (0.87-1.02) |  |  |
|  |  | 21.5g/day median | 0.98 (0.90-1.06) |  |  |
|  |  | 35.6/day median | 1.04 (0.96-1.12) |  |  |

IS, ischemic stroke; HS, hemorrhagic stroke; BMI, body mass index, WHR, waist to hip measurement ratio; HDL-C, high density lipoprotein cholesterol.
